# One Health: A Strategic Paradigm Shift to Propel Iran’s Vaccine Industry into its Second Century

**DOI:** 10.34172/aim.34326

**Published:** 2025-10-01

**Authors:** Zahra Baradaran-Seyed

**Affiliations:** ^1^Razi Vaccine and Serum Research Institute, Agricultural Research, Education and Extension Organization (AREEO), Karaj, Iran

 Iran recently celebrated the centenary of its vaccine industry, marking a significant achievement in public health and biomedical innovation. Over the past century, the country has established a robust infrastructure for producing vaccines and therapeutic serums, supported by key public institutions and international collaborations.^1^ The Pasteur Institute of Iran, established in 1920, and the Razi Vaccine and Serum Research Institute, founded in 1924, have played a crucial role in combating infectious diseases in humans and animals. Their contributions have positioned Iran as a leader in vaccine production in the Middle East and approximately tenth worldwide. This highlights the significant importance of sustainable vaccine development in enhancing public health and global health initiatives.^1,2^

 The latter half of the initial century posed significant challenges for Iran’s vaccine sector. Several studies have highlighted the pressing need for enhanced research and development (R&D) through public-private partnerships (PPP), which have led to various spin-offs and the establishment of private companies.^2^ However, critical issues such as workforce training and financial support have yet to be adequately addressed during this transition.^2,3^ The COVID-19 pandemic has necessitated a comprehensive reevaluation of priorities, particularly concerning the nation’s manufacturing capabilities. This shift has prompted stakeholders to emphasize the development of infrastructure. Nonetheless, a considerable challenge persists in effectively addressing the fundamental issues surrounding the R&D of vaccines targeting emerging infectious diseases, particularly in time-sensitive scenarios and under resource constraints.^2^

 Iran’s continued dependence on imported vaccines highlights a critical vulnerability in its public health infrastructure. As the source countries have developed their vaccine industries after Iran, it is imperative to prioritize the establishment of a robust and innovative domestic vaccine production system.^3^ Nonetheless, the obstacles related to human resources, vaccine manufacturing enterprises, and financial backing (particularly in the context of forming PPP) are just a few of the challenges that must be addressed.

 In the aftermath of the September 11 attacks involving anthrax as a bioterrorism agent, there was a notable shift in the global landscape of public health and security. Emerging threats prompted a reassessment of strategies and protocols to safeguard global health security.^4^ Zoonotic pandemics and public health emergencies such as severe acute respiratory syndrome (SARS), highly pathogenic avian influenza (HPAI), Ebola, and COVID-19 have revealed vulnerabilities in health systems and prompted greater investment in vaccine R&D.^5^

 In light of these shifts, a promising path for Iran’s vaccine industry as it enters its second century lies in embracing the One Health approach. This holistic paradigm integrates human, animal, and environmental health to combat zoonotic diseases.^6^ This approach acknowledges that approximately 80% of bioterrorism agents and 75% of novel infectious pathogens originate from animal reservoirs. Zoonotic diseases result in an estimated 2.7 million annual deaths and 2.5 billion illnesses worldwide. The initiative promotes transdisciplinary collaboration among veterinarians, clinicians, ecologists, and policymakers to develop and implement policies that prevent spillover events from animal hosts. It also addresses environmental issues intensified by climate change, such as habitat loss, to create comprehensive countermeasures.^7,8^

 A primary objective of One Health Vaccinology is to prevent the transmission of zoonotic diseases. Effective immunization programs (illustrated by initiatives aimed at controlling rabies in domestic dogs and wildlife) have been shown to reduce human rabies cases by up to 90%.^6^ Such programs highlight the importance of addressing diseases in animal populations to prevent their spillover to humans. Collaborative vaccination strategies are vital, particularly in responding to outbreaks, such as HPAI that impacted U.S. dairy cattle during the 2024-2025 H5N1 outbreaks.^9^ These events highlight the importance of a comprehensive approach that considers the inter-species relationships of pathogens to mitigate risks to both human and animal health.^7,8^ In addressing antimicrobial resistance, One Health Vaccinology promotes the reduction of disease incidence through effective vaccination, thereby decreasing reliance on antibiotics.^6,8^ This proactive approach is essential in combating a significant global health threat. Protecting biodiversity is another critical aspect of this framework, as the development of species-specific vaccines, including innovative oral formulations for wildlife, plays a vital role in managing diseases like rabies in fox populations.^6^ Sustainable vaccine manufacturing practices are also key to advancing development efforts. Employing green technologies, such as plant-derived vaccines for infections like influenza and Ebola, can help minimize ecological footprints associated with traditional vaccine production, benefiting public health while contributing to environmental conservation amidst ongoing habitat degradation.^10^ Timely detection of pathogens in wildlife, insects, and ecological habitats is crucial for developing effective countermeasures against these threats. Early identification enables rapid responses to potential outbreaks, ensuring the protection of both animal and human health. Evolving regulatory frameworks, including expedited emergency use approvals and mechanisms such as the U.S. Food and Drug Administration’s (FDA’s) Animal Rule, facilitate advanced vaccine design, enabling the swift translation of robust animal trials into timely human applications.^5^ The rapid response to the 2024 Marburg virus outbreak in Rwanda exemplifies the effectiveness of these adaptive regulatory processes.

 In conclusion, to usher Iran’s vaccine industry into its second century, adopting the One Health paradigm is critical, as it addresses the interconnected health of humans, animals, and the environment. Embracing a One Health approach will not only foster innovation but also strengthen the resilience of the vaccine industry, ensuring sustainable public health advancements for future generations of the countries. Global partnerships with organizations such as the World Health Organization (WHO), the World Organization for Animal Health (WOAH, founded as OIE), and the Food and Agriculture Organization (FAO), as well as collaborative networks, are crucial for advancing vaccine R&D. These collaborations enable countries to effectively address public health challenges through innovative approaches that strengthen health systems. Improving diagnostic laboratories and upgrading biosafety level facilities will enhance early detection of pathogens, particularly for zoonotic diseases. Integrating the One Health Surveillance System with vaccine development, aligned with WHO, WOAH, and FAO standards, will facilitate the creation of advanced vaccines for both human and animal populations.^6,7^ Improving R&D budget and infrastructure, and enhancing human resource training, in alignment with the One Health collaboration, will drive innovation, reduce import dependency, and improve market competitiveness, thereby positioning Iran as a regional leader in sustainable vaccine development.
